# Making Clinic Letters Make Sense: A Patient-Focused QI Project

**DOI:** 10.1192/bjo.2026.11516

**Published:** 2026-06-30

**Authors:** Emily Wing, Nicholas Strouther, Anna Watkin

**Affiliations:** 1 Mersey Care NHS Foundation Trust, Liverpool, United Kingdom; 2 Cheshire and Wirral Partnership NHS Foundation Trust, Chester, United Kingdom

## Abstract

**Aims:**

Clinic letters are a key method of communication between secondary mental health services, primary care, and patients. However, traditional psychiatry clinic letters are often lengthy, clinician-centred, and difficult for readers to quickly identify key information and actions. This sits at odds with national priorities emphasising patient-centred care, shared decision-making, and improved collaboration across healthcare interfaces.

Informal feedback within our service suggested that existing clinic letters lacked clarity and accessibility for both general practitioners (GPs) and patients.

This quality improvement project aimed to design and implement a structured, patient-addressed clinic letter template and to evaluate its impact on satisfaction with letter length, layout, ease of understanding, and content among GPs and patients.

**Methods:**

A structured clinic letter template was developed through multidisciplinary discussion, informed by best-practice guidance and principles of patient-centred communication. Key features included clear headings, concise language, explicit action points, and written directly to the patient, with GPs copied into correspondence.

GP satisfaction with clinic letters was assessed before and after implementation using a brief feedback survey. Respondents rated satisfaction with letter length, layout, ease of understanding, and content as satisfied, neutral, or dissatisfied. Following implementation, patient feedback was also collected, as patients did not routinely receive clinic letters prior to this project.

**Results:**

GP satisfaction was assessed pre- and post-intervention (n=6 pre; n=8 post); patient feedback was collected post-intervention (n=12).

Following implementation of the structured clinic letter template:

- GP satisfaction with*letter length*increased from17% to 100%.
- GP satisfaction with*letter clarity*increased from50% to 100%.
- GP satisfaction with*letter content*increased from33% to 87%(neutral responses reduced from 50% to 0%).
- GP satisfaction with*layout*increased from17% to 88%(neutral responses reduced from 50% to 0%).

Patient feedback demonstrated 100% satisfactionwith letter length, clarity, content, and layout.

Routine sharing of clinic letters with patients represented an additional improvement post-intervention, improving transparency and patient involvement.

**Conclusion:**

Implementation of a structured, patient-addressed clinic letter template led to substantial improvements in GP and patient satisfaction with letter length, clarity, content and layout. This project demonstrates how simple, low-resource changes to written communication can support patient-centred care and improve collaboration across the primary–secondary care interface, aligning with national strategic priorities for mental health services.

